# Silent Dangers: The Growing Vulnerability of Older Adults in Fatal Road Traffic Accidents

**DOI:** 10.7759/cureus.80712

**Published:** 2025-03-17

**Authors:** Stefania Ungureanu, Camelia-Oana Muresan, Veronica Ciocan, Raluca Dumache, Emanuela Stan, Georgiana-Denisa Gavrilita, Alexandra Enache

**Affiliations:** 1 Discipline of Forensic Medicine, Bioethics, Deontology, and Medical Law, Department of Neuroscience, Victor Babeş University of Medicine and Pharmacy, Timișoara, ROU; 2 Legal Medicine, Timișoara Institute of Legal Medicine, Timișoara, ROU; 3 Ethics and Human Identification Research Center, Victor Babeş University of Medicine and Pharmacy, Timișoara, ROU; 4 Doctoral School, Victor Babeş University of Medicine and Pharmacy, Timișoara, ROU; 5 Genetics, Timișoara Institute of Legal Medicine, Timișoara, ROU

**Keywords:** fatal, medico-legal autopsy, older adults, road traffic accidents, vulnerable road users

## Abstract

Introduction: Older adults represent a growing and vulnerable road user group. Our aim was to develop targeted interventions and preventive measures for decreasing road deaths in older adults by establishing the circumstances and the characteristics leading to such fatalities with regard to each type of road user.

Materials and methods: Medico-legal autopsy records of victims of road traffic accidents from Timișoara Institute of Legal Medicine (TILM), Romania, in a five-year period (2017-2021) were analyzed. Data was retrieved and grouped according to the victims’ demographics (age and gender), date of the accident, type of road user (driver, passenger, pedestrian, cyclist), and traumatic injuries. Moreover, data from the traffic police reports regarding additional circumstances of the crash were retrieved.

Results: A total of 51 medico-legal autopsies involving older adults (aged above 70 years) were performed at TILM in 2017-2021. Regarding the type of road user, pedestrians were the most affected group among older adults (n=17), followed by cyclists (n=13), passengers (n=11), and drivers (n=10). Most cases (n=9) occurred when the victims were hit by a car behind them. The second most seen circumstance that led to fatally injured older victims was represented by being hit by a moving vehicle while crossing the street outside of a marked crosswalk (n=7).

Conclusion: Our findings illustrate the need to focus on road fatalities among older adults, as these tragic events affect this vulnerable group. Unfortunately, less attention has been given to the severity and significance of these incidents. Preventive campaigns focusing on road user education, safe driving, safe walking and the implementation of facile public transport or improved pedestrian crossings should be the key points.

## Introduction

Road traffic accidents (RTAs) are a well-known global problem, due to the injuries, disabilities, and fatalities they cause. According to the 2023 World Health Organization (WHO) report on road safety, road crashes represent the 12th leading cause of death when all ages are considered. Moreover, 69% of road deaths occur among people of working age but 23% occur among people aged 60 years or above [1,2]. Senior road fatalities in Europe in 2022, according to the European Commission, represent 29% of all road fatalities. Additionally, with an average of roughly 64 senior fatalities per one million senior citizens in the European Union road traffic, seniors have the second-highest mortality rate of any age group [3].

Being independent and active for as long as feasible is essential for healthy aging in all facets of life [4]. Therefore, driving a car can be considered one of the requirements for healthy aging, but it may also compromise the safety of senior drivers [5]. It is true that senior drivers pose a unique risk in the context of traffic, particularly when it comes to fatal RTAs [6]. Older drivers significantly affect other road users by raising the risk of morbidity and death for their passengers as well as the occupants of other cars [7]. A large number of serious and fatal collisions involve elderly pedestrians; specifically, 29% of senior fatalities in 2022 were pedestrians [3,8]. The likelihood of severe injuries rises with pedestrian age (the chance of fatality increases nearly exponentially with age) [9,10]. Road fatalities in older adults occur either because they are more likely to be injured due to their increased frailty or because they are more likely to crash in certain situations brought on by aging (e.g. diminished motor, cognitive, and sensory abilities such as eyesight or certain medical conditions) [11-13]. Additionally, numerous medications often taken by adults over 65 may impair one's ability to drive, such as sedative-hypnotics, antihypertensives, antidepressants, and hypoglycemic medicine [14].

The elderly represent a growing and vulnerable road user group [15]. The overall number of senior road users, whether as pedestrians or car occupants, has increased in tandem with the aging population [7]. There are several situations to consider when promoting safe driving to age healthily and addressing them will call for a range of preventive actions [6]. In fact, compensatory behaviors like slowing down are insufficient to stop traffic accidents once a certain degree of psychomotor deficit has been attained (alteration of cognitive function, with particular emphasis on executive functions, as measured by neuropsychological tests, and also attitude and self-reported driving behavior) [16]. A step in this direction is to study and understand the risk factors that cause road fatalities in older adults.

According to European statistics, Romania had the highest mortality rate for seniors per million people per country in 2022. The mortality rate for Romania was 119.8 in comparison to the European mean of 63.6 [3]. According to these statistics, road injuries among the elderly in this region may be a major health issue that requires extra care. Therefore, we believe it is important to analyze the circumstances that result in such fatalities and to formulate preventive measures. 

Medico-legal autopsies are mandatory in Romania for all victims of RTAs to determine the cause and manner of death, time since death, and circumstances of death [17]. By analyzing medico-legal autopsy reports, important information can be obtained that can be used in public campaigns aimed at reducing road deaths among all age groups.

There is ongoing discussion on the proper age at which geriatric care should begin. Although most research includes defined older individuals using a cut-off age of 65, some studies also utilize a lower cut-off age of 60 [18]. In line with the study done by Ungureanu et al. on medico-legal autopsies in a five-year period in Western Romania, we considered older adults having the age above 70 years old. This age group represented 12.9% of victims of fatal RTAs in the five-year period studied (2017-2021) [19].

Our aim was to develop targeted interventions and preventive measures for decreasing road deaths in older adults. Seeing that existing preventive measures are driver-oriented, and the emphasis of road user education is on safe driving conduct, an analysis of the circumstances and the characteristics leading to fatalities in older adults, regarding each type of road user, not only focused on the driver’s aspects, was needed. We expected to encounter most road accidents to be related to maneuvers performed by the older driver, with fewer cases attributed to other types of road users. We assessed the monthly distribution of cases, age and gender-based variations, the types of road users, and the traumatic injuries the victims sustained in the accidents. Moreover, we assessed the circumstances in which the road accidents happened, according to the traffic police reports. This study comes as an addition to the article published by Ungureanu et al. that deals with road fatalities in children, in which we illustrated the risk factors related to fatal RTAs in children aged 0-17 [19]. 

The burden of all road fatalities emphasizes the need for the identification of risk factors and prevention strategies to reduce the important global crisis in all the victims [19]. Numerous studies have demonstrated that even in collisions of comparable intensity, older adults had higher rates of death and morbidity than younger ones [20]. This disparity can be explained by aging-related physical and mental deficits [7].

We believe that data obtained from medico-legal autopsy records in countries that conduct such autopsies for every road fatality can provide valuable insights into the circumstances of RTAs. Furthermore, this information can support the creation of targeted interventions and campaigns to reduce road fatalities among older adults across all types of road users. In this study, our objective was to assess the conditions in which fatal RTAs occur in older adults to develop targeted campaigns aimed at reducing road fatalities in older adults for all types of road users. To the best of our knowledge, this is the first study that focuses on older adults as victims of fatal RTAs in the Romanian population to develop protective strategies and preventive measures by establishing the circumstances that led to the accidents. This study will bring new evidence to the literature on risk factors involved in RTAs in older adults.

## Materials and methods

This was a retrospective analysis of medico-legal autopsy records from January 1, 2017, to December 31, 2021, from the archives of Timișoara Institute of Legal Medicine (TILM), Timișoara, Romania, to determine the circumstances and characteristics leading to fatal RTAs. The study was approved by the Scientific Research Ethics Committee of Victor Babeş University of Medicine and Pharmacy (approval number: 88/19.12.2022 rev 2024) and was conducted in accordance with the Declaration of Helsinki.

Inclusion and exclusion criteria

Medico-legal autopsy records at TILM of adults aged above 70 years old who were victims of fatal RTAs between 2017 and 2021 were included. Medico-legal autopsy records of people aged less than 70 years, who were not victims of RTAs, and records not pertaining to the period mentioned in the inclusion criteria were excluded.

Data collection

Data from the autopsy records was retrieved and grouped according to the victims’ demographics (age and gender), date of the accident, type of road user (driver, passenger, pedestrian, cyclist), traumatic injuries sustained in the accidents, and the cause of death. Circumstances regarding the accidents were retrieved from the traffic police reports that are kept together with the autopsy records in the archives.

Procedure of record keeping

In Romania, when a person dies in a car accident, the traffic police send a report to TILM in which they ask the legal medicine doctor to perform the autopsy and answer questions related to the cause and manner of death, traumatic injuries sustained by the victim, whether the victim died due to the traumatic injuries that happened in the accident, blood alcohol concentration (depending on the case). This traffic police report also offers information regarding the characteristics and the circumstances leading to the accident and what type of road user the victim was. The circumstances of the accident can be described in detail depending on the police officer who wrote the report or the type of report they used. These traffic police reports and the medico-legal autopsy records are kept together in the archives. These are physical documents stored in the archives and are not digitalized. Therefore, we manually extracted the data from these documents, and we looked in the police reports for all the information we could find regarding additional circumstances of the crashes.

Data analysis

The fatal RTAs of older adults were manually collected and introduced into an Excel spreadsheet (Microsoft Office 365 suite; Microsoft Corporation, Redmond, Washington, United States). The items followed were the number of the autopsy, the age and gender of the victim, the date of the accident, the date of death, the type of road user (driver, pedestrian, cyclist, passenger), and the traumatic injuries. The item retrieved from the traffic police reports is the circumstances in which the accident occurred. These were then analyzed using simple mathematical tools and descriptive statistics (mean, median, standard deviation, and data visualization such as pie charts, bar charts, and histograms). The data was grouped according to the victims’ demographics (age and gender), age groups, age groups and gender, date of the accident, type of road user (driver, passenger, pedestrian, cyclist), type of road user, gender of the victim, type of road user and age of the victim, traumatic injuries, and cause of death. As the sample was relatively small, no statistical analysis was conducted.

## Results

During the five-year study period, a total of 51 medico-legal autopsies involving older adults (aged above 70 years) were performed at TILM. The total number of all medico-legal autopsies in 2017-2021 was 3752 and 1.35% (n=51) of them were represented by older adults that died in RTAs. They also represent 12.9% of all 395 medico-legal autopsies of victims of fatal RTAs.

Monthly variation

**Figure 1:**
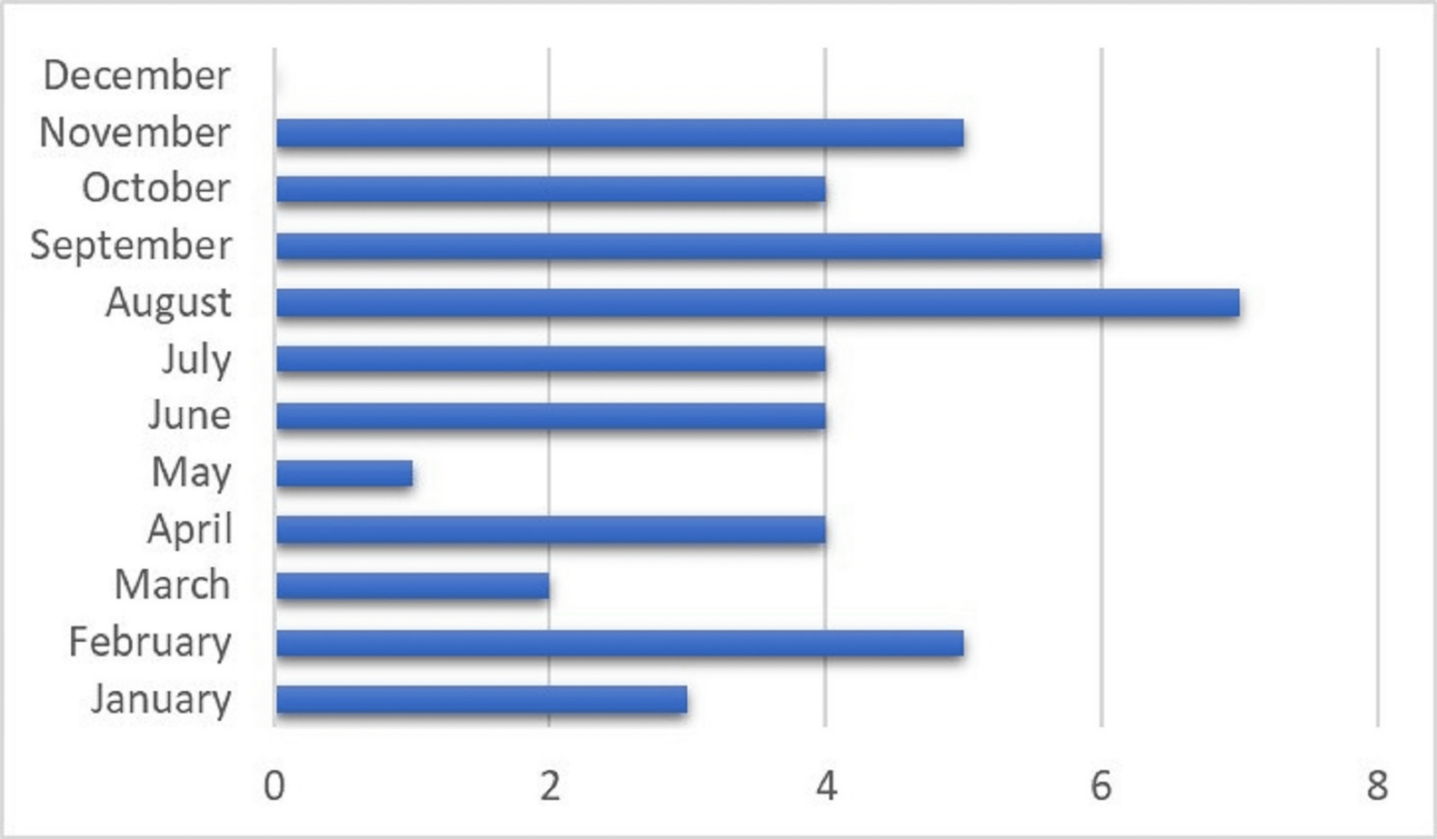
Monthly variation of road fatalities in older adults. Data is presented as number.

The monthly distribution of fatal RTAs in older adults is represented in Figure 1. As illustrated, the highest number of cases was seen in August (n=7, 13.7%) while in December we did not encounter any fatal road crashes among this age group.

Age and gender variations

In adults aged >70 years who were fatally injured in RTAs, the male-to-female ratio was 2:1 (34 male and 17 female victims). The ages of the victims ranged from 71 years to 95 years with a mean age of 77.3 ± 5.83 years. The mean age of male victims was 77.14 ± 5.61 years while for females, it was 77.70 ± 6.22 years. We further stratified age into age groups as follows: 71-75 years, 76-80 years, 81-85 years, 86-90 years, and above 90 years. Table 1 illustrates the distribution of victims according to age groups.

**Table 1:** Distribution of older adults into age groups in 2017-2021 (N=51)

Age groups (years)	71-75	76-80	81-85	86-90	>90
Number of victims	25	12	9	3	2

**Figure 2:**
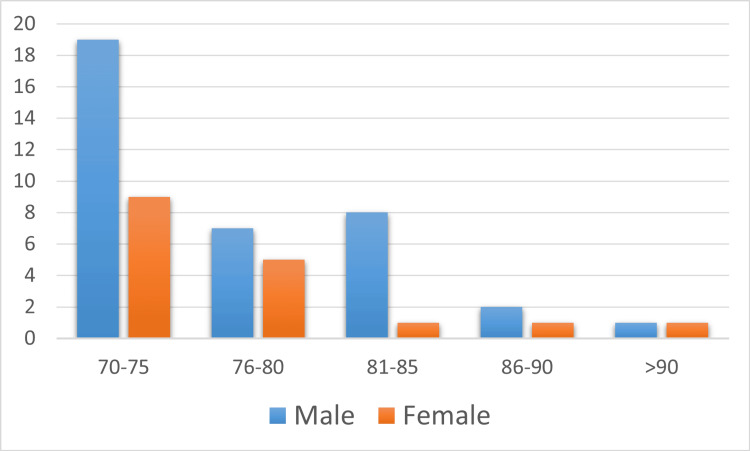
Age and gender variations of road fatalities in older adults in 2017-2021 (N=51) Data is presented as number.

The gender variations according to age groups can be seen in Figure 2, which illustrates the sociodemographic data of the victims.

Types of road user

**Figure 3:**
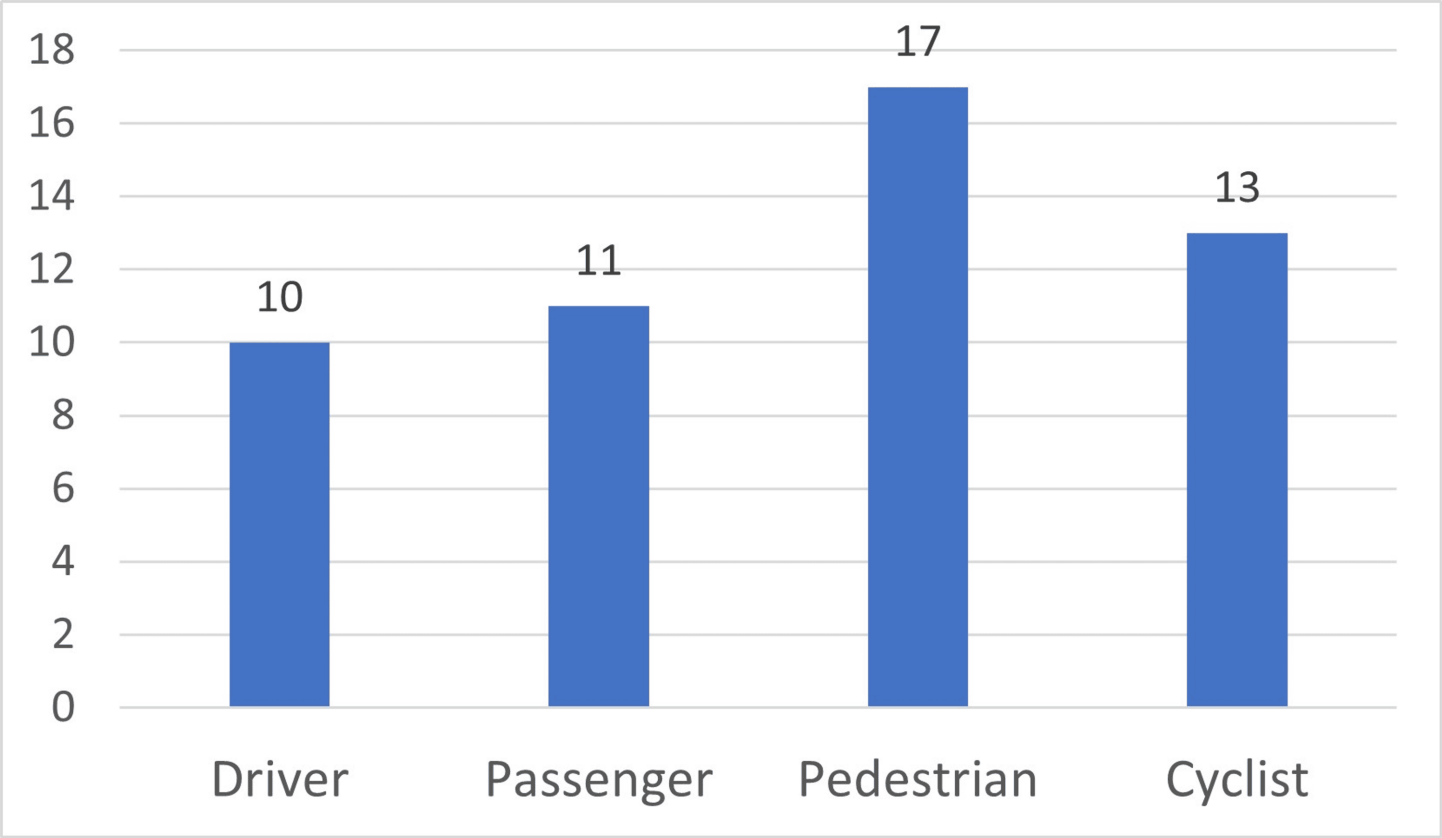
The distribution of road fatalities in older adults according to the type of road user (N=51) Data is presented as number.

Pedestrians were the most affected group among older adults that died in RTAs (n=17; 33.33%), followed by cyclists (n=13; 25.5%), passengers (n=11; 21.6%), and drivers (n=10; 19.6%). Figure 3 illustrates the distribution of victims according to the type of road user.

**Figure 4:**
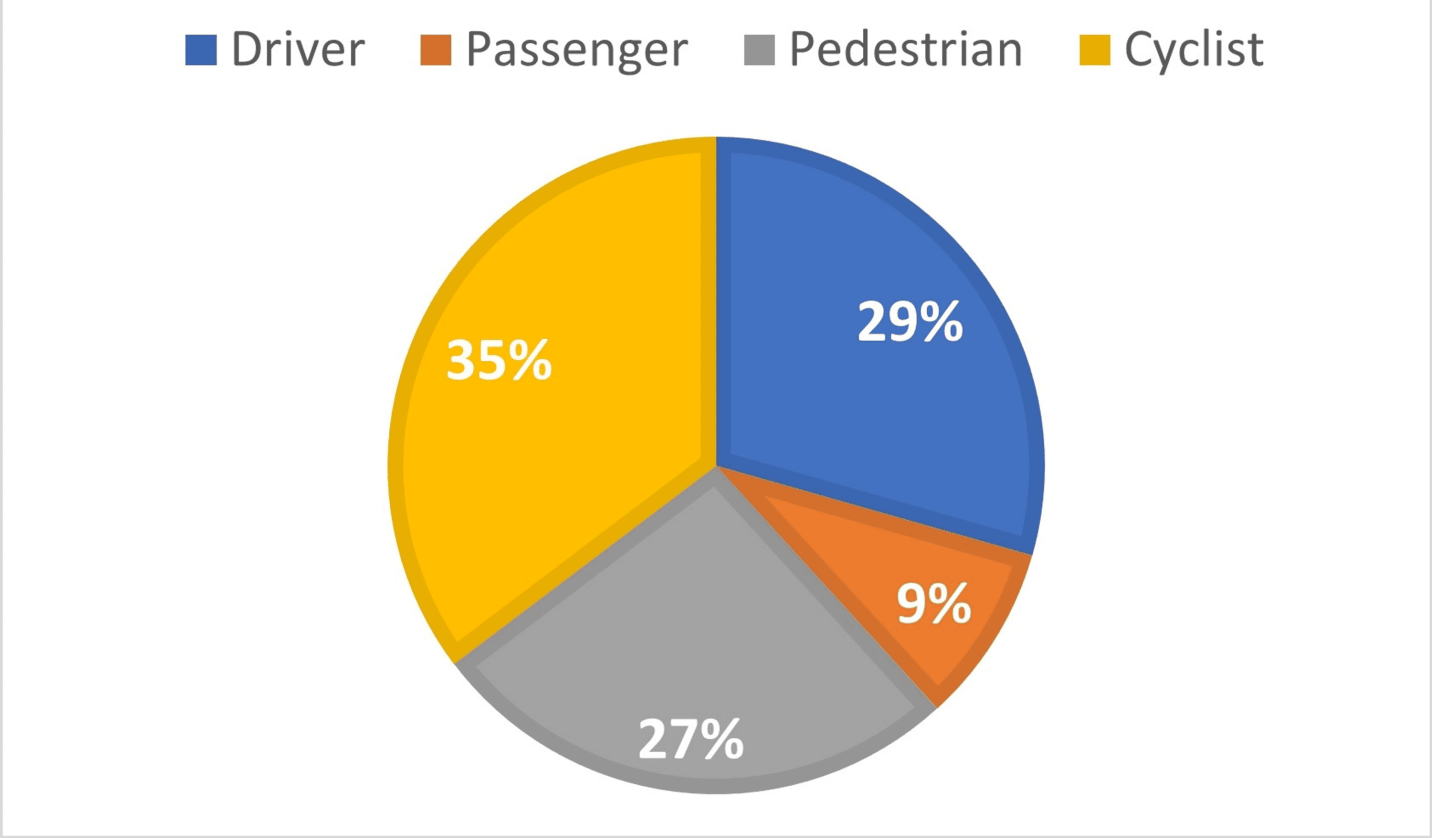
Distribution of road fatalities in older adults according to the type of road user for male victims.

**Figure 5:**
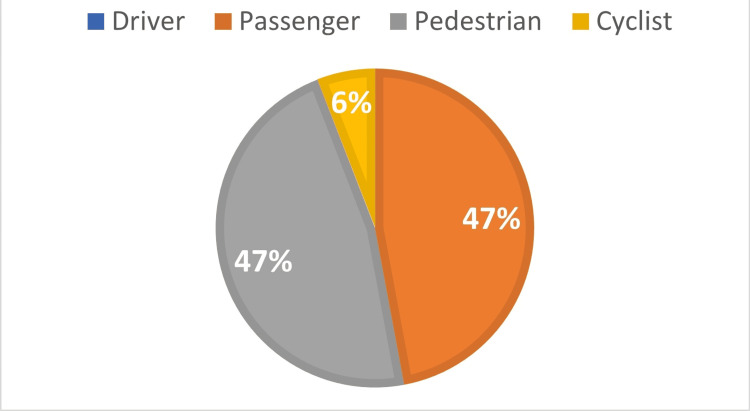
Distribution of road fatalities in older adults according to the type of road user for female victims.

When we further stratified the victims according to their gender and the type of road user, we noticed that all the drivers (n=10) were males, most passengers were females (n=8), and most of the cyclists were males (n=12). The distribution of the victims regarding their type of road user in relation to gender is given in Figure 4 and Figure 5.

Circumstances in which fatal RTAs happened

Analyzing all fatal RTA cases involving older adults over a five-year period allowed us to identify the accident circumstances. However, in 19 cases (37.2%), we did not find any relevant information about how the RTAs happened, other than specifying that the victim was involved in a RTA and the type of road user. Table 1 represents an elaborate analysis of the circumstances that led to fatal RTAs in which the older adults died.

**Table 2:** Circumstances of fatal RTAs in older adults by analyzing medico-legal autopsy records in a five-year period (2017-2021) RTA: road traffic accident

Circumstance	Number of cases	Type of road user			
		Driver	Passenger	Pedestrian	Cyclist
Frontal collision between two cars	4	-	+	-	-
Hit by the car from behind	7	+	-	-	+
Hit by a car while crossing the street at a crosswalk	4	-	-	+	-
Hit by a car while crossing the street outside of a marked crosswalk	5	-	-	+	-
Hit by a bus while crossing the street outside of a marked crosswalk	1	-	-	+	-
Hit by a tram while crossing the street outside of a marked crosswalk	1	-	-	+	-
Losing control of the car when not respecting the speed limit, and hitting a bridge head or a ditch on the side of the road	5	+	+	-	-
Collision between two cars while entering traffic	1	+	-	-	-
Hit by a car that was back-driving while being on the sidewalk	1	-	-	+	-
Hit by a car while the pedestrian fell on the road	1	-	-	+	-
Hit by the car behind them while veering	2	-	-	-	+
Hit by a car while not stopping at a stop sign	1	-	-	-	+
Horse-carriage hit by a car	1	+	-	-	-
No information available	19	+	+	+	+

As illustrated above, in most cases (n=9; 17.6%), the victims were hit by a car from behind them. The victim was either a driver that was hit by a car behind them (n=1) which means a collision between two cars, or was a cyclist (n=8). In the latter case, the cyclist was either going carefully while the car behind hit and fatally injured them (n=6) or the cyclist did not take precautions while veering left and was hit by a car that was driving behind them (n=2). In two cases, the guilty driver left the crash site.

The second most seen circumstance that led to fatally injured older victims was being hit by a moving vehicle (a car, bus, or tram) while crossing the street outside of a marked crosswalk (n=7; 13.7%). All such victims were pedestrians.

The third most seen circumstance in fatally injured older adults was represented by losing control of the car when not respecting the speed limit and hitting a bridgehead or a ditch on the side of the road (n=5). In this scenario, the victims were either the guilty drivers (n=3) or passengers either in the same vehicle as the drivers or in another one.

A frontal collision between two cars was seen in four cases, while one of the cars was overtaking another car (n=3; 5.8%) or when a malfunction of the steering system led to a frontal collision. In all these cases, the fatally injured victim was a passenger.

Pedestrians were hit by a car while crossing the street at a crosswalk in four cases.

Traumatic injuries and cause of death

**Figure 6:**
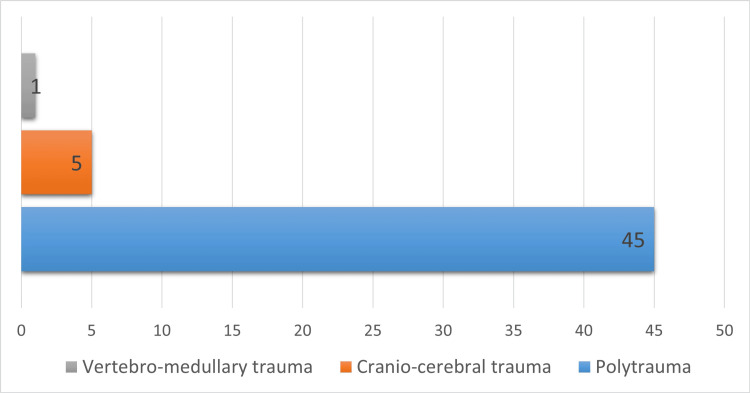
Cause of death of the victims Data presented as number.

When investigating the cause of death sustained by older adults in fatal RTAs, we noticed that most of them presented polytrauma (n=45) (Figure 6).

The most common traumatic injuries are illustrated in Table 3. An association of these injuries resulted in polytrauma for most of the victims. In addition, fractures of various bones in the upper limbs (10 cases), lower limbs (11 cases), and pelvic fractures (11 cases) were also sustained by some victims.

**Table 3:** Most common traumatic injuries sustained by the victims.

Type of trauma	Classification of trauma	Frequency (Percentage)
Cranio-cerebral trauma	Skull fractures	19 (37.2%)
	Subdural/extradural hematoma	23 (45.1%)
	Subarachnoid hemorrhage	32 (62.7%)
	Cerebral contusions and lacerations	20 (39.2%)
Vertebro-medullary trauma	Atlanto-occipital dislocation	3 (5.9%)
	Cervical body fractures	11 (21.6%)
	Thoraco-lumbar body fractures	19 (37.2%)
Thoracic trauma	Rib and sternal fractures	40 (78.4%)
	Lung contusions and lacerations	21 (41.2%)
	Hemothorax	28 (54.9%)
	Tears of thoracic blood vessels or heart muscle	9 (17.6%)
Abdominal trauma	Hepatic tears and lacerations	10 (19.6%)
	Splenic tears and lacerations	7 (13.7%)
	Renal tears and lacerations	4 (7.8%)
	Intestinal trauma	1 (1.9%)

## Discussion

The number of active older adults on roads and corresponding deaths are the most alarming issues given the aging of the population. To address the anticipated demographic shift, it is imperative from a public health standpoint to start raising national awareness of road safety planning. The high incidence of RTAs and fatalities among the elderly stems from inadequate road safety measures for this vulnerable group, while current policies remain largely ineffective [18].

Romanian autopsy-based studies offer an epidemiological profile of road accidents, seeing that all road fatalities need to have a medico-legal autopsy performed. Data derived from autopsy findings can support the future implementation of suitable preventive actions. Our findings illustrate that older adults accounted for 12.9% of all road fatalities in 2017-2021. In the systematic review and meta-regression analysis by Ang et al., RTAs’ overall pooled prevalence estimates among senior citizens were 14.0% and the highest mortality rates were found among pedestrians and elderly people aged 74 years and above [18]. In Spain in 2018, the fatality rate for the elderly was more than three-fold the rate for the rest of the victims of road crashes [21]. However, our results are not in accordance with this study. We believe this is because the majority is represented by the productive age groups which are more vulnerable to fatal RTAs, because older adults may have less risky driving behavior, they drive less during nighttime or rush hour time, and they have more experience. Nonetheless, this group of people needs to be protected as they are vulnerable road users, and they are more likely to suffer serious injuries [15].

In the present study, by analyzing fatal RTAs over a five-year period, we observed that more than half of them were related to a maneuver performed by the older driver. This is in accordance with findings by Skyving et al. on what triggers road traffic fatalities among older adult drivers [6]. Moreover, this is in line with other global studies. A study from the United Kingdom attributed 52% of the fatalities among drivers aged 60 and over to the driver's own actions [22]. Moreover, studies from Florida (United States) [23] and Australia [24] found even higher proportions for drivers aged 65+, 64% and 67%, respectively. One common description of older drivers is that they have a high traffic fatality rate. Both a higher collision participation rate and more severe injuries can contribute to this high death rate [7]. According to our study, pre-existing medical conditions of the driver had no bearing on road crashes, since every medico-legal autopsy also consists of performing microscopic examination of tissue samples. According to a regional study from Munich, Germany, that used autopsy and police information, only 2% of driver fatalities were caused by disease [25].

Road accidents in older adults do not only put the driver at risk. This is also in accordance with our findings, in which the driver represented the least involved victim (10 cases) being surpassed by all the other types of road users (11 passengers, 13 cyclists, and 17 pedestrians). This does not suggest that preventive measures should not be oriented toward drivers because most of these fatalities occurred in a collision with a car in which the driver can be held accountable, except for the pedestrians that were crossing the street outside of a marked crosswalk or the cyclists that were veering, disregarding the traffic behind them. For senior bikers, intersections and other complicated traffic situations can be particularly challenging to navigate. Due to physical limitations, older cyclists may exhibit more erratic behavior and may also choose a certain move at the last minute [26]. A study done by Etehad et al. on the impact of road traffic accidents on the elderly, demonstrated that pedestrians and motorcycle riders had a substantially higher death rate than other victim types [7]. They found that the percentage of pedestrian occurrences rose noticeably according to increase in age. According to Kim and Ulfarsson, better street crossing conditions and intersections are essential to promoting senior pedestrian safety because older people are more likely to be involved in collisions with turning cars [27]. However, a study conducted in Australia revealed that motor vehicle occupants, not pedestrians, were primarily responsible for the fatality rate [13]. Yet, according to a systematic review and meta-analysis on the epidemiology of road traffic injuries among elderly people, the most common type of traffic injury victim among the elderly was pedestrians (48.1%) [28].

An important key factor in achieving road safety is represented by road user education to avoid accidents [21]. Actions aimed at creating age-friendly environments, such as enhancing public transport and promoting walking for older adults, are being addressed. However, special attention must be given to promoting safe driving, as older individuals also rely on driving [29]. Thompson et al. propose measures such as reduced speed limits and divided sealed roads, which are advantageous for all ages of drivers' safety [30]. In addition, although the effectiveness of these required measures is still being questioned, several nations require medical screenings and driving skills tests for older drivers when they reapply for licenses to accommodate their functional changes [31-33]. Transportation alternatives need to be considered if the person is not fit to drive [21]. Additionally, according to studies, pedestrian-vehicle collision victims have two times the odds of dying compared to victims of collision between motor vehicles, indicating that greater focus on road safety and education measures is necessary for this group [7,18,34]. Preventive strategies such as pavements, speed bumps, and road safety laws need to be addressed to ensure road safety [18]. Improvement of pedestrian crossings such as a better design of traffic islands or traffic signals, improvement of cyclist infrastructure, and improvement of intersection designs are the main measures proposed by the Transport Research Centre in the Czech In-Depth Accident Study on seniors in road traffic [35]. The focus when discussing measures to reduce RTAs among the elderly is mostly driver-oriented, with senior pedestrians receiving less attention [28]. The study done by Rivera-Izquierdo et al. on the prevention of road crashes in older adults brings attention to the role family physicians play in the prevention of road crashes among older adults since this age group represents the population that most frequently visits health centers [21]. Doctors could advise patients on their driving abilities, for instance, or whether they should give up driving altogether if they are unfit to do so.

We believe that data derived from medico-legal autopsy records in a country that performs a medico-legal autopsy on every road fatality may bring to attention new information regarding the circumstances of RTAs. Moreover, it may help develop targeted interventions and campaigns aimed at reducing road fatalities in older adults for all types of road users.

We must take into consideration the limitations of our study. First, in 19 cases (37.2%) it was not possible to pinpoint a crash trigger, which was due to the lack of information in the traffic police reports regarding the circumstances of the accidents. This aspect may hinder a comprehensive understanding of accident triggers. If driving-related issues, such as distraction, had played a role in those incidents, deaths from self-inflicted maneuvers would have risen. Another limitation is the lack of information about pre-crash comorbidities and underlying illnesses of the victims. However, these deaths cannot be disease-related, since the autopsy and the microscopy performed on tissue samples did not reveal any pathologies that would explain the crashes. Another limitation is the relatively small sample of cases, because of which statistical analysis was not done. This may affect the generalizability of our findings. However, we still consider this study highly relevant in today’s environment seeing that Romania leads in the number of senior road fatalities. In this context, more studies regarding the factors that lead to road fatalities need to be done to be able to pinpoint the circumstances involved in RTAs to identify prevention tactics.

For future research, it is important to analyze RTAs that do not result in fatalities in this population. This will help identify the causes of crashes for different types of road users, especially for older victims who are injured but not killed in RTAs.

## Conclusions

Our findings illustrate that most of the road deaths in older adults happen due to maneuvers related to drivers; however, these tragedies affect all types of road users. Moreover, pedestrian maneuvers such as crossing the street outside of a marked crosswalk represent an important crash trigger. Specific measures such as reduced speed limits and implementing safe driving conduct without any distractions or impairments should be the objectives of campaigns aimed at educating the public. Medical screenings and driving skills tests for older drivers in the context of reapplying for a driving license may be of help. Pavements, speed bumps, and road safety laws should be implemented as prevention strategies to promote road safety. Additionally, improvement of pedestrian crossings, cyclist infrastructure, and intersection designs need to be addressed. Ultimately, transportation alternatives should be available for seniors who are no longer able to drive. Thus, preventive campaigns focusing on road user education, safe driving, safe walking, and the implementation of facile public transport or improved pedestrian crossings should be the key points.
